# Nonlinear two-level dynamics of quantum time crystals

**DOI:** 10.1038/s41467-022-30783-w

**Published:** 2022-06-02

**Authors:** S. Autti, P. J. Heikkinen, J. Nissinen, J. T. Mäkinen, G. E. Volovik, V. V. Zavyalov, V. B. Eltsov

**Affiliations:** 1 Low Temperature Laboratory, Department of Applied Physics, Aalto University, POB 15100, FI-00076 Aalto, Finland; 2 Department of Physics, Lancaster University, Lancaster, LA1 4YB UK; 3 Department of Physics, Royal Holloway University of London, Egham, Surrey TW20 0EX UK; 4 L.D. Landau Institute for Theoretical Physics, Moscow, Russia

**Keywords:** Quantum fluids and solids, Magnetic properties and materials, Quantum mechanics, Bose-Einstein condensates

## Abstract

A time crystal is a macroscopic quantum system in periodic motion in its ground state. In our experiments, two coupled time crystals consisting of spin-wave quasiparticles (magnons) form a macroscopic two-level system. The two levels evolve in time as determined intrinsically by a nonlinear feedback, allowing us to construct spontaneous two-level dynamics. In the course of a level crossing, magnons move from the ground level to the excited level driven by the Landau-Zener effect, combined with Rabi population oscillations. We demonstrate that magnon time crystals allow access to every aspect and detail of quantum-coherent interactions in a single run of the experiment. Our work opens an outlook for the detection of surface-bound Majorana fermions in the underlying superfluid system, and invites technological exploitation of coherent magnon phenomena – potentially even at room temperature.

## Abstract (web summary)

Recent work has reported a realization of a time crystal in the form of the Bose-Einstein condensate of magnons in superfluid ^3^He. Here, the authors study the dynamics of a pair of such quantum time crystals and show that it closely resembles the evolution of a two-level system, modified by nonlinear feedback.

## Introduction

Perpetual ground state motion in equilibrium defines a time crystal, but observing such motion is famously unfeasible^1^. Experimental time crystal realisations thus bend either the equilibrium^2–4^ or the perpetuity^5–7^ requirement, reaching stability only if isolated from the environment and the observer^1,8–10^. Consequentially, coupling separate time crystals while retaining sufficient isolation is challenging, and time crystals have yet not been studied in a dynamic environment. We arrange spontaneous two-level dynamics of interacting time crystals, each consisting of 10^12^ magnons, in the superfluid B phase of ^3^He (^3^He-B). In this system, the observable time crystal life time can be extended up to a thousand seconds^11^ (10^9^ periods of motion) in the absence of a driving force, while the underlying superfluid system provides intrinsic feedback for engineering coherent dynamics.

Magnons in ^3^He-B arise as the quanta of transverse spin waves, associated with magnetisation that precesses about the external magnetic field **H**. At sufficient magnon density and low enough temperature, the precession synchronises spontaneously at uniform frequency *ω* and phase, forming a magnon Bose–Einstein condensate^12–14^. The spontaneous synchronisation can be demonstrated by pumping magnons to a higher energy level in the confining trap from which they spontaneously fall to the ground state^5,15^, or even by pumping incoherent magnons to the system using a noise drive^7,16^. This shows that the magnon state in the BEC is decoupled from the drive. The transverse spin precession of the magnon condensate, therefore, manifests the characteristic spontaneous periodic motion of a time crystal^5,6^.

The time crystal can be created using two different pumping techniques. Using a continuous drive yields a Floquet (discrete) time crystal. Here we use the pulsed technique where the drive is turned off before the time crystal evolution begins. This approach allows us to study uncontrived time crystal dynamics and interactions in the absence of external enforcement. The time crystal formation during the pumping pulse and its evolution thereafter is characterised by two timescales. The first timescale *τ*_*E*_ ~ 0.1 s describes the time crystal thermalisation^17^, that is, how quickly the precession becomes coherent at the ground level in a trap, following the pumping of magnons. The second timescale *τ*_*N*_ is the time crystal lifetime. In an isolated sample container *τ*_*N*_→*∞* exponentially as temperature decreases. In practice there are also losses in the circuit that is coupled to the precessing spins for control and observation purposes. It is therefore necessary to allow for a finite *τ*_*N*_. The time crystal remains well defined as long as *τ*_*N*_ ≫ *τ*_*E*_^5,6^.

**Fig. 1:**
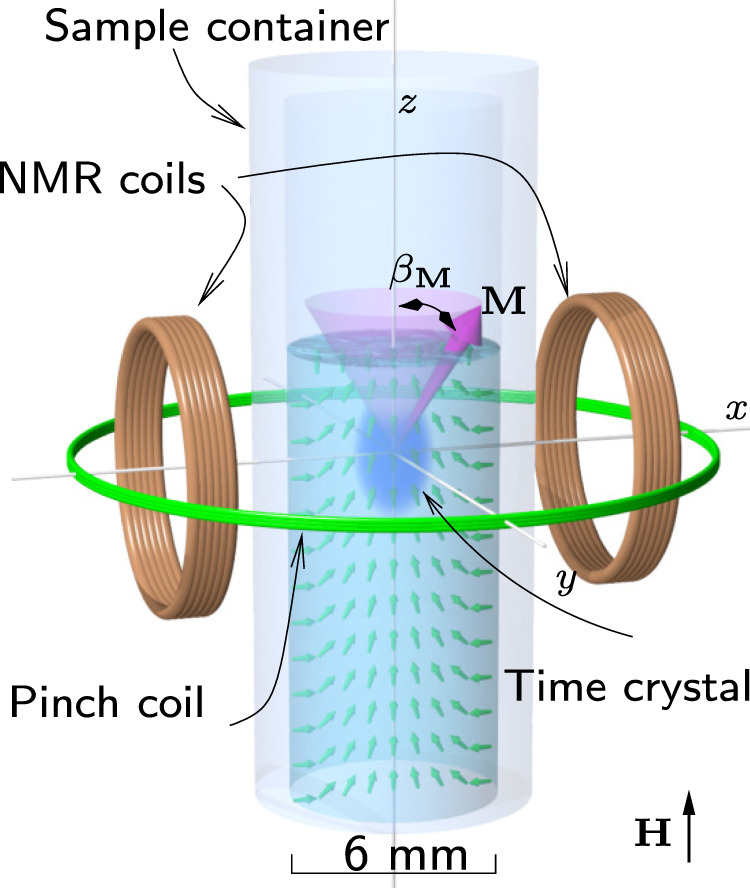
Schematic illustration of the experiment. The superfluid ^3^He sample is contained in a quartz glass cylinder. The magnon time crystal (blue blob) is trapped in the middle of the container by the combined effect of a minimum in the static magnetic field, created using a pinch coil (green wire loop), and by the spatial distribution of the superfluid orbital momentum **L** (small green arrows). The coherent precession of magnetisation **M** (magenta cone) in the time crystal is observed using transverse pick-up coils. The static magnetic field **H** is oriented parallel to the axis of the cylinder. The ripple on the superfluid free surface is added for illustrational purposes. The two-level time crystal is schematically illustrated in Fig. 2.

**Fig. 2:**
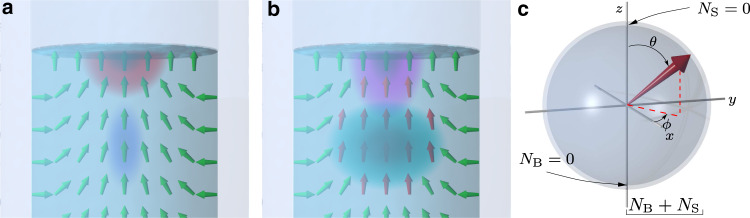
Time crystal two-level system. **a** The distribution of **L** (green arrows) confines magnons in two local minima, hosting two adjacent time crystals: one in the bulk of the superfluid (blue blob) and the other one touching the free surface (red blob). In each time crystal, magnetisation is precessing coherently, which couples to measurement circuitry as shown in Fig. 1. **b** Magnons in the bulk modify the confining trap created by the **L** distribution. When the bulk population is large (cyan blob), the textural trap is widened (red arrows), which modifies also surface time crystal’s wave function (magenta blob). This increases the coupling between the states. Changes in the trap and the wave functions have been exaggerated for illustrational purposes. **c** The state of the two-level system (red arrow) can be illustrated using a Bloch sphere where radial distance corresponds to magnon number *N*_B_ + *N*_S_, the relative phase between the time crystals’ precession corresponds to the azimuthal angle *ϕ*, and the polar angle *θ* describes the relative weights of the two-level basis states in the “superposition” (see Methods).

In superfluid ^3^He, Cooper pairs possess orbital momentum whose average distribution, parametrised by vector **L**, is axially symmetric in the sample container (Fig. 1). The time crystal is trapped in the middle of the superfluid sample by that distribution owing to spin-orbit interaction. We fine-tune the trap by adding a magnetic field profile as detailed in Methods^18–20^. We also place a free surface of the superfluid above the bulk trap centre^6^. The free surface distorts the distribution of **L** as shown in Fig. 2a, resulting in a second local minimum, located 3 mm above the bulk trap minimum. Magnons can be trapped and form time crystals in either of the traps or both of them simultaneously. In this Article we concentrate on the lowest energy level in each trap. We denote the time crystals “bulk” and “surface” corresponding to the physical location. The location of a time crystal is identified from experimental records by its response to changes in the magnetic field profile^6^.

Let us denote the bulk time crystal population (that is, the number of trapped magnons) *N*_B_ and the surface population *N*_S_. The bulk and surface precession frequencies (*ω*_B_, *ω*_S_) are determined by the profile of the confining trap, and the coupling Ω between the crystals by the overlap of their wave functions, as detailed in Methods. We will show that the dynamics of the coupled levels are described by the macroscopic two-level Hamiltonian

1 $${{{{{\mathcal{H}}}}}}=\hslash \left({\begin{array}{lc}{\omega }_{{{{{\rm{B}}}}}}[{N}_{{{{{\rm{B}}}}}}(t)]&-{{\Omega }}\\ -{{\Omega }}&{\omega }_{{{{{\rm{S}}}}}}\end{array}}\right),$$

where *ℏ* is the reduced Planck constant and *t* is time. Note that this two-level system is conveniently parametrised by a macroscopic Bloch sphere (Fig. 2c) with the relative precession phase between the time crystals corresponding to the azimuthal angle and level populations corresponding to projection on the *z* axis, in a direct analogy with the Bloch sphere description of microscopic two-level systems such as qubits. In what follows we measure all frequencies in the frame rotating at the Larmor frequency *ω*_0_ = ∣*γ***H**∣ taken at the centre of the bulk trap (*γ* ≈ −2⋅10^8^rad s^−1^ T^−1^ is the ^3^He gyromagnetic ratio).

The essential difference of Eq. (1) from a standard two-level Hamiltonian is the dependence *ω*_B_[*N*_B_(*t*)], which arises due to a nonlinear feedback provided by the spin-orbit trap: a large local magnon density expands the trap, changing the distribution of **L**, thus decreasing *ω*_B_. This mechanism is extensively studied in refs. ^5,13,14,21^, and the outcome is schematically illustrated in Fig. 2b. Near the free surface the **L** distribution is fixed perpendicular to the surface. Therefore *ω*_S_ is constant to a good approximation. As the time crystal populations decay, *N*_B_ decreases and *ω*_B_ increases. We can thus make the energy levels in the double trap cross by selecting suitable initial populations. A rigorous description of the macroscopic time crystal wave functions, the trapping potential, and the feedback mechanism are derived in Methods. We emphasise that all the relevant technical explanations can be found in Methods also where not explicitly referenced.

We note that another characteristic feature of time crystals, the lack of heating under continuous drive^22^, is also manifest in this system. Under continuous pumping, the number of magnons in the time crystal is determined by the chemical potential that corresponds to the pumping frequency (see Methods). When this number would be exceeded, the time crystal spontaneously decouples from the drive^13^, thus preventing overheating. Similarily, during the slow population decrease after a pumping pulse, the chemical potential (precession frequency) is continuously adjusted to the changing magnon number^21^. Hence, the precession period and coherence become incommensurate with and thus independent of the drive pulse even if the drive was originally resonant.

In this Article, we study the dynamics of the two-level time crystal system, carrying out two experiments. In the first experiment, where the level crossing takes place at small *N*_B_, we showcase crossing dynamics that follow the textbook description: the system is initially in the ground state, but at an avoided level crossing both levels are populated owing to Landau-Zener population transfer. This “superposition” state continues to be modified by Rabi population oscillations after the crossing. The second experiment starts from a “superposition”, the level crossing takes place at large *N*_B_ where the feedback mechanism transforms into dynamically changing coupling Ω. Analysis of the second experiment shows that co-existing time crystals lay many-body interactions bare for the capable observer in a single run of the experiment. That is, in this case, the level crossing dynamics cannot be described analytically, but while coherent quantum phenomena are often hidden from direct inspection, time crystals have no such limitations.

## Results

### Basic two-level dynamics at small *N*_B_

**Fig. 3:**
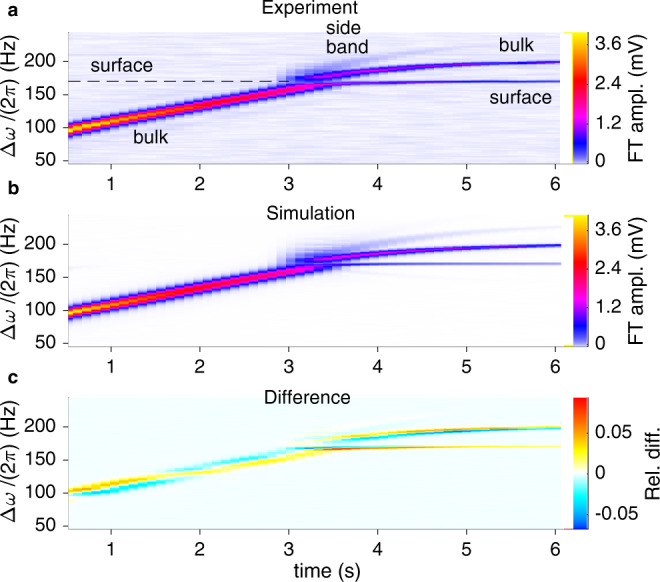
Two-level time crystal dynamics at small *N*_B_. **a** The signal from the pick-up coils, analysed with windowed Fourier transformation (FT), shows the bulk time crystal as a moving sharp peak. Frequency is plotted in the rotating frame Δ*ω* = *ω* − *ω*_0_. The excitation pulse at *t* = 0 is framed out for clarity. Initially *ω*_B_ < *ω*_S_, but as the population in the bulk trap decays, at *t* = 3.3 s, the global ground state moves to the surface in an avoided crossing. The excited state, now located in the bulk, is simultaneously populated. Rabi (Josephson) population oscillations are seen as a side band. Coupling extracted from the side band extrapolates to Ω/(2*π*) = 1.7 ± 0.4 Hz at the crossing, in good agreement with the fitted simulation value Ω/(2*π*) ≈ 1.4 Hz. **b** The numerical simulation recreates the population transfer and the side band, confirming that the population transfer is due to Landau-Zener transition (analysis in Fig. 4). In the absence of measurement noise also the side band of the surface time trace is weakly visible. **c** Subtracting the simulation from the experiment shows that the point-wise residuals remain smaller than 5%. The relative difference is normalised by the total signal at the crossing. In this measurement temperature was 180 μK and *ω*_0_/(2*π*) = 833 kHz.

**Fig. 4:**
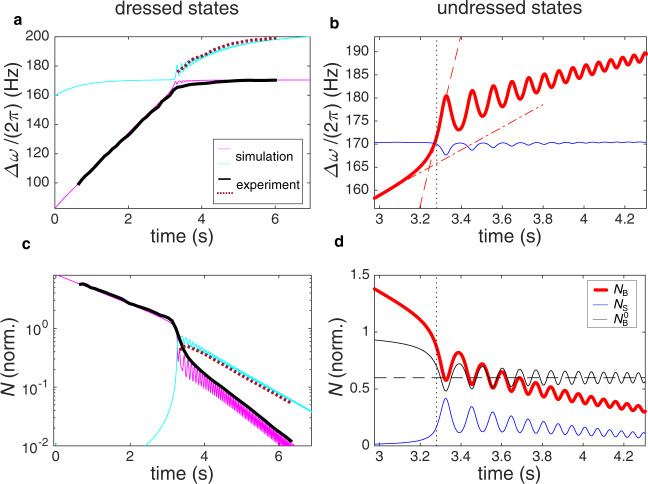
Analysis of two-level dynamics at small *N*_B_. **a** The dressed ground level time crystal frequency in the simulation (thin magenta line) follows that extracted from the experiment (thick black line). The experimental line is obtained by tracing the maximum in the Fourier spectrum shown in Fig. 3 (oscillations are filtered out by the long time window). The ground state frequency initially corresponds to *ω*_B_, and after the avoided crossing at *t* = 3.3 s it corresponds to *ω*_S_. Simultaneously, the excited level frequency (simulation - thin cyan line, experiment - dotted thick dark red line) switches from *ω*_S_ to *ω*_B_. Oscillations of the frequencies after the crossing arise owing to the population oscillations. **b** We can hence use the simulation to extract the undressed frequencies *ω*_B_ (thick red line) and *ω*_S_ (thin blue line), which cross at *t* = 3.3 s (dotted vertical line). The crossing rate d∣*ω*_S_ − *ω*_B_∣/d*t* ≈ d*ω*_B_/d*t* is accelerated intrinsically by the feedback *ω*_B_(*N*_B_), as illustrated by the different slopes of the dash-dotted and dashed red lines. The Landau-Zener population transfer magnitude is determined by this speed-up. Frequencies are plotted in the rotating frame Δ*ω* = *ω* − *ω*_0_. The corresponding populations are shown in the panels below: **c** The measured signal amplitude (ground level - thick black line, excited state - dotted thick dark red line) agrees with the simulated dressed populations (ground level - thin magenta line, excited state-thin cyan line). Both the total population from simulation and the measured signal are normalised to one at the crossing, and the filling factors applied for comparison in Fig. 3 (see Methods) are not used here. **d** The undressed populations *N*_B_ (thick red line) and *N*_S_ (thin blue line) are extracted from the simulation. The total population is normalised to one at the crossing. The black line shows the bulk population with the exponential decay compensated, ${N}_{{{{{{{{\rm{B}}}}}}}}}^{0}={N}_{{{{{{{{\rm{B}}}}}}}}}\exp (t/{\tau }_{{{{{{{{\rm{B}}}}}}}}})/{N}_{{{{{{{{\rm{B}}}}}}}}}(t=0)$. After the crossing, the compensated bulk population averages at ${N}_{{{{{{{{\rm{B}}}}}}}}}^{0}=0.61$ (horizontal dash line), corresponding to the fraction of population transferred to the excited state by the Landau-Zener mechanism.

The time crystal levels can be populated in desired proportion by a radio-frequency pulse via adjacent coils (Fig. 1). To highlight the two-level dynamics, we populate only the bulk time crystal in the beginning of the experiment shown in Fig. 3a. After the pulse, the coherent precession of magnetisation induces an oscillating signal in the coils, which allows inferring the precession frequency, and the signal amplitude yields the magnon number. These quantities are extracted from the experiment in Fig. 4. The pumping is followed by exponential decay of ${N}_{{{{{{{{\rm{B}}}}}}}}}(t)={N}_{{{{{{{{\rm{B}}}}}}}}}(t=0)\exp (-t/{\tau }_{{{{{{{{\rm{B}}}}}}}}})$ with time constant *τ*_B_, controlled by temperature as detailed in Methods.

In Fig. 3, the ground level is initially located in the bulk trap, *N*_B_ decays at a rate determined by *τ*_B_, and *ω*_B_ increases slowly as the trap recovers a narrower shape. Meanwhile, *ω*_S_ remains constant. Hence, *ω*_B_ eventually crosses *ω*_S_ before levelling out. In a coupled two-level system, a level crossing has specific consequences: The observed frequencies are the (dressed) eigenfrequencies of the Hamiltonian which deviate from the undressed frequencies *ω*_B_, *ω*_S_ in the Rabi regime Ω > ∣*ω*_B_ − *ω*_S_∣. Due to this hybridisation, the observed levels avoid crossing each other, and the global ground level smoothly switches from bulk to surface (from *ω*_B_ to *ω*_S_) as seen in Fig. 3a. Population transfer between the levels is also observed, as both of the levels are populated after the avoided crossing.

Traversing the avoided crossing adiabatically would allow the entire magnon population to follow the global ground state, but here some magnons move to the state with higher eigenenergy (precession frequency). This process is generally known as Landau–Zener–Stueckelberg–Majorana tunnelling, or Landau–Zener tunnelling. The population transferred depends on the rate of level crossing d∣*ω*_S_ − *ω*_B_∣/d*t* at *ω*_B_ = *ω*_S_. For usual coupled non-linear oscillators with damping, the crossing rate is determined directly by the damping. Here the decay rate *τ*_B_ = 3.5 s corresponds to d∣*ω*_B_[*N*_B_(*t*)]/(2*π*)∣/d*t* ~ 24 Hz s^−1^. Using this and the coupling directly extracted from the experiment as explained below yields the predicted Landau–Zener population transfer fraction 0.9% to the excited state, which is two orders of magnitude smaller than that observed in the experiment. In this case, only one of the two levels would be visible in Fig. 3a after the crossing. In similar experimental runs with smaller d∣*ω*_S_ − *ω*_B_∣/d*t* we have observed population transfer up to 20 orders of magnitude larger than the corresponding Landau-Zener prediction.

We can analyse this striking mismatch by simulating the time evolution of the two-level Hamiltonian numerically. We feed the experimentally-determined bulk and surface decay rates, *τ*_B_ and *τ*_S_, the corresponding initial populations, and the measured *ω*_B_[*N*_B_] dependence to a numerical simulation of the two-level Hamiltonian (see Methods). The coupling constant Ω is used as a fitting parameter yielding Ω/(2*π*) = 1.4 Hz.

The outcome of the simulation, plotted in the same way as the experimental signal, is shown in Fig. 3b. We can directly compare it with the experiment by subtracting the simulated time-dependent Fourier spectrum from the experimental one (Fig. 3c). The simulation underestimates the change of the surface time crystal frequency near the crossing as shown in Fig. 4a,b, which causes the largest deviation between the Fourier spectra. Otherwise the typical deviation between the two signals, as normalised by the total signal at the crossing, is less than 5%. In particular, the simulation replicates the magnitude of the population transfer, that is, 60% of magnons move to the excited state. The simulated population dynamics are compared directly with the measured signal in Fig. 4c. We emphasise that repeated runs of the simulation with perturbed input parameters reveal that this qualitative level of population transfer is insensitive to the precise value of any of the input parameters.

To explain this observation, we extract the undressed frequencies from the simulation in the region near the avoided crossing (Fig. 4b). As a result of the feedback *ω*_B_(*N*_B_), the bulk frequency is changing both owing to the slow decay of *N*_B_ and because of the population transfer from *N*_B_ to *N*_S_ by Rabi oscillations. Thus, their combined effect increases the crossing rate d∣*ω*_S_−*ω*_B_∣/d*t*. The magnitude of the Landau-Zener population transfer is determined within ~50 ms of the crossing^23^, and in this window *ω*_B_(*t*) can be linearised. Inserting the accelerated crossing rate to the Landau-Zener formula yields the expected population transfer of 61%, in good agreement with the simulated population transfer, 60% (Fig. 4d). That is, the population transfer follows the Landau-Zener description with the crossing rate taken at the instant of level crossing. Note that the Landau-Zener description is thus valid even if the crossing rate is regulated by intrinsic feedback. We conclude that the observed population transfer strongly supports the two-level interpretation of the time-crystal dynamics.

Far from the avoided crossing, the two-level interaction is characterised by AC Josephson population oscillations between the levels^6^. Owing to the feedback in the bulk trap, the oscillations result in a side band that follows the bulk trace. The frequency of the population oscillations is set by the difference of the time crystal precession frequencies, equal to the difference of their chemical potentials^6^. Thus, the side band is separated from the bulk trace by ∣*ω*_B_−*ω*_S_∣, as seen in Fig. 3a (see derivation in Methods). The amplitude of the population oscillations is determined by the coupling Ω, and the relative side band amplitude by the slope of *ω*_B_(*N*_B_) (formulas are given in Methods). Thus, we can extract the coupling directly from the experimental data, yielding Ω/(2*π*) = 1.7 ± 0.4 Hz in the level crossing region, in good agreement with the simulation fitted value Ω/(2*π*) = 1.4 Hz.

Put together, the Josephon population oscillations, the Landau–Zener population transfer, and the agreement on the two-level coupling independently extracted from the different aspects of the population dynamics confirm that the two-time crystals form a macroscopic two-level system.

### Dynamical coupling regime at large *N*_B_

**Fig. 5:**
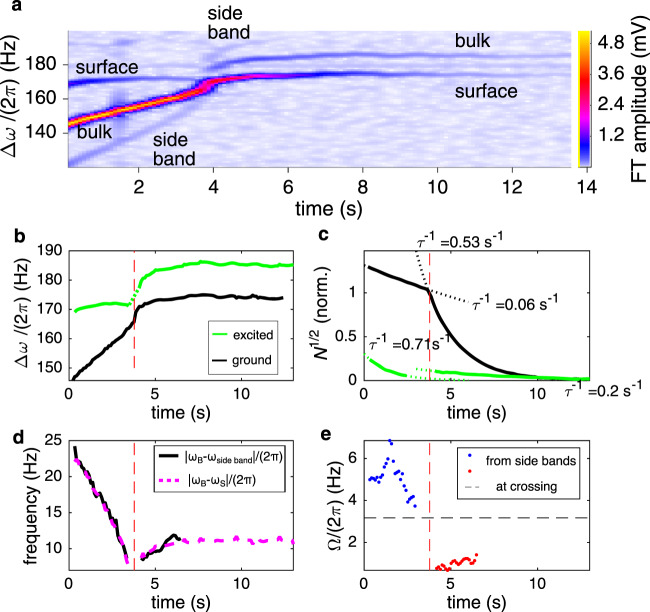
Two-level time crystal with dynamic coupling at large *N*_B_. **a** The time crystals are created at *t* = 0. Frequencies are plotted in the rotating frame Δ*ω* = *ω* − *ω*_0_. **b** Initially the ground level (black line) is located in the bulk and the excited level (solid green line) at the surface. At *t* ≈ 3.8 s (vertical dash lines), the ground level moves smoothly to the surface in an avoided crossing. Dotted green line shows a linear interpolation of the excited-state frequency at the crossing. **c** Most of the population follows the ground level (black line) movement from bulk to surface, identified by a sharp increase in the exponential relaxation rate *τ* (fitted dash lines and values marked in the figure). Total population is normalised to one at the crossing. **d** Population oscillations between the time crystals are seen as a side-band of the bulk crystal trace in panel **a** at frequency *ω*_side band_. The side band’s frequency separation from the bulk trace, ∣*ω*_B_ − *ω*_side band_∣ (solid black line), is equal to the frequency separation of the main traces, ∣*ω*_B_ − *ω*_S_∣ (magenta dash line). **e** The coupling Ω can be extracted from the side band and main trace amplitudes, in good agreement with that estimated by linear interpolation from the separation of the main traces in panel **b** (horizontal dash line). In this measurement, temperature was 150 μK and *ω*_0_/(2*π*) = 624 kHz.

Magnon time crystal dynamics, enhanced by the nonlinear feedback, can be analysed also directly without resorting to a numerical simulation of the system. This is advantageous as it will allow untangling interactions involving multiple time crystals that go beyond the two-level description. As a simple demonstration of this capability, we introduce a level crossing in a region where *N*_B_ is an order of magnitude larger than above. The resulting trap modification affects not only *ω*_B_(*N*_B_) but also the constriction between the time crystals, as sketched in Fig. 2b. This causes the coupling Ω to change dynamically in the course of the crossing. Both levels are populated in the beginning of the experiment (Fig. 5a) to allow following their dynamics directly.

In this experiment the coupling is changing, but qualitatively the dynamics follow a similar pattern as above: The ground state moves from bulk to surface when the undressed frequencies cross at *t* ≈ 3.8 s (Fig. 5b). The moment of the crossing is identified by a sharp increase in the ground state trace relaxation rate from ${\tau }_{{{{{{{{\rm{B}}}}}}}}}^{-1}=0.06\,{{{{{{{{\rm{s}}}}}}}}}^{-1}$ to ${\tau }_{{{{{{{{\rm{S}}}}}}}}}^{-1}=0.53\,{{{{{{{{\rm{s}}}}}}}}}^{-1}$ (Fig. 5c). The increase is attributed to increased dissipation in the surface trap due to surface-mediated emission of other spin wave modes^24^ and potentially surface-bound Majorana states^25,26^, but a detailed study is left for the future. We note that the states can be identified also by adjusting the magnetic field profile and rerunning the experiment; the relaxation rate is a convenient shortcut for distinguishing the two levels.

Josephson population oscillations between the two time crystals are seen as the side band that follows the bulk time crystal trace. As explained above, the side band is separated from the bulk trace by ∣*ω*_B_ − *ω*_S_∣. This separation is characteristic of the Josephson effect, and it changes in time because *ω*_B_ changes, as shown in Fig. 5d. The surface time crystal is not followed by a similar side band, because the surface trap is rigid and hence population oscillations result in no side bands (see Methods). A second bulk trace side band should be located symmetrically on the other side of the bulk trace, but it exactly coincides with the surface trace and is therefore not resolvable.

The side band amplitude allows us to extract the coupling between the time crystals (Fig. 5e). The extracted coupling is the largest in the beginning of the experiment and decreases when *N*_B_ decreases. That is, the constriction between the time crystals is affected by the bulk trap modification (Fig. 2b), which makes the coupling larger when *N*_B_ is large. This is qualitatively in line with the trap modification mechanism discussed in refs. ^13,14,21^.

Near the avoided crossing interference effects prohibit direct access to the population oscillation in the experiment. However, in a two-level system the coupling can also be extracted from the minimum frequency separation of the dressed frequencies of the two levels at the avoided crossing, which is equal to 2Ω. This is done by interpolation in Fig. 5a. The result is shown by the horizontal line in Fig. 5e, in good agreement with the dependence extracted from the side bands. Note that near the avoided crossing the separation of the dressed (observed) frequencies equal to the difference between the Josephson frequency and the Rabi frequency. That is, Rabi population oscillations smoothly replace the Josephson oscillations, increasing the population oscillation frequency as compared with the Josephson frequency, which goes to zero.

It is worth noting that the relaxation rate of the bulk time crystal depends on whether it is the global ground state or the global excited state. In Fig. 5c the bulk relaxation time is ${\tau }_{{{{{{{{\rm{B}}}}}}}}}^{-1}=0.06\,{{{{{{{{\rm{s}}}}}}}}}^{-1}$ until the level crossing (ground state), and it increases to ${\tau }_{{{{{{{{\rm{B}}}}}}}}}^{-1}=0.2\,{{{{{{{{\rm{s}}}}}}}}}^{-1}$ after the level crossing (excited state). The same observation, correspondingly, applies to the surface time crystal relaxation rates. We emphasise that such change is never observed in the absence of the level crossing, for example, if the bulk time crystal is the ground state throughout the experiment. That is, the excited state seems to slowly leak magnons to the ground state. This observation hints that there is an additional incoherent channel that allows magnons to move from the excited state to the ground state, showing independently that the two time crystals interact and that the level crossing has physical consequences that penetrate the dynamics in the two-level system.

The above analysis confirms that the two-level description is valid and robust against dynamic variation its parameters, and that direct experimental observations provide continuous access to all relevant aspects of the interaction.

## Discussion

To summarise, we have shown that the dynamics and interactions of the two adjacent magnon time crystals are quantitatively described by a two-level Hamiltonian. The levels are modified by a nonlinear feedback, arising owing to spin-orbit interaction in the underlying superfluid system. This allows engineering intrinsic time crystal dynamics in the absence of continuous external drive. We show that when the two-level eigenfrequencies approach one another, the coupling between the levels results in an avoided crossing with ensuing Landau-Zener population transfer from the global ground state to the excited state. Rabi population oscillations, combined with the feedback mechanism, increase the population transfer by orders of magnitude. This is quantified by comparing numerically simulated population dynamics with the experiments. We also show that all relevant observables and parameters including the eigenfrequencies and the coupling between the time crystals can be simultaneously extracted from the experiment. We emphasise that each measurement sequence shown in this Article corresponds to a single run of the experiment, but the phenomena are well reproducible.

We have shown that the spin-orbit interaction can be harnessed to create a nonlinear feedback for magnons in a coherent time crystal system. Nonlinear feedback is needed for spin-based versions of quintessential quantum devices such as the SQUID. It remains an interesting task to explore the time-crystal two-level system further by demonstrating parametric pumping of magnons and logic gate operations between the two levels. For example, parametric pumping can be arranged by modulating the magnetic part of the trap at frequency Ω. Additionally, any number of co-existing time crystals can be accommodated in a magnetic landscape to increase the number of degrees of freedom, and the flexible trap can be turned off by adjusting the external magnetic field. These are important capabilities for realising magnon-based devices^27–32^. To access phenomena such as quantum entanglement, few-magnon operations can be implemented using nano-fluidic confinement and ultra-sensitive NMR techniques^33,34^. We emphasise that similar physical phenomena including quasiparticle Bose-Einstein condensation and the emergence of time crystals can be accessed in certain solid-state room-temperature systems, for example based on magnons in YIG films^35–42^. This opens the outlook of quasiparticle-based coherent on-chip applications in ambient conditions, including coherent quantum information processing^27,28,30–32,39^.

The three-dimensional topological superfluid is wrapped by a two-dimensional system of surface-bound quasiparticles, among them Majorana fermions^43–49^. At the free surface there are no impurities (unlike at sample container walls), and surface-bound Majorana fermions are expected to manifest themselves as detectable zero-temperature magnetic dissipation^25,26^. Majorana fermions have remained elusive despite a decade of searching in different condensed matter systems^50^. The hybridised two-level state is in direct contact with the free surface of the superfluid, making and extremely sensitive probe for the bound Majorana states. We have provided preliminary evidence that the surface induces magnetic dissipation and that details of this signature can be explored using the time-crystal two-level system.

## Methods

### Experiment

The superfluid ^3^He sample is placed in a cylindrical quartz-glass container (15 cm long, 6 mm diameter) in a nuclear demagnetisation refrigerator (Fig. 1). The lower end of the sample container connects to a volume of sintered silver powder surfaces, thermally linked to the nuclear refrigerant. This allows cooling the ^3^He down to 130 μK. Temperature of the superfluid is measured using a quartz tuning fork^51,52^, and pressure is equal to saturated vapour pressure, which is vanishingly small at these low temperatures. The superfluid transition temperature at saturated vapour pressure is *T*_c_ ≈ 0.9 mK. The sample container is surrounded by two transverse NMR coils, which are part of a tank circuit resonator with *Q* ≈ 150, and a pinch coil used to create an axial minimum of the magnetic field. The resonance frequency of the tank circuit can be tuned in eight equidistant steps between 550 kHz and 833 kHz, corresponding to external magnetic fields between 16.5 mT and 25 mT. The signal is amplified by a cold preamplifier^53^ and room temperature amplifiers.

The free surface is located 3 mm above the centre of the magnetic field minimum. The location of the free surface is adjusted by removing ^3^He slowly until the desired location is achieved, measuring the pressure of ^3^He gas in a calibrated volume that results from the removal of liquid from the originally fully filled sample container. The outcome is favourably compared with the observed magnon spectrum and a numerical model of the trap. The resulting two traps for magnons are detailed in the next Section.

The time crystal wave function can be written as Ψ = *ae*^−*iωt*^, where *t* is time, *ω* is the precession frequency related to the chemical potential *μ* = *ℏω*, the phase term *e*^*iφ*^ is contained in *a*, and the number of magnons *N* = ∣*a*∣^2^. The tipping angle of the precessing magnetisation *β*_**M**_, measured from the magnetic field **H**, parametrises the spatial profile of the wave function, $N=| a{| }^{2}\propto \int {\sin }^{2}\frac{{\beta }_{{{{{{{{\bf{M}}}}}}}}}}{2}{{{{{{{\rm{d}}}}}}}}V$. The signal induced in the pick-up coils (Fig. 1) is sinusoidal, corresponding to the magnetisation along the axis of the NMR coil, or in other words, real part of the rotating complex wave function, *e*^−*iωt*^. The measured signal amplitude is proportional to the amplitude of the time crystal wave function,

2 $$A=c\sqrt{N},$$

where *c* contains the so-called filling factor of the state within the NMR coils, the amplification provided by the tank circuit resonator and other amplifiers in the measurement circuit^53^, and physical constants^20,21^.

A desired level in the trap can be populated by a radio-frequency pulse via the pick-up coils, followed by slow population decay owing to two mechanisms: The fermionic thermal excitations of the superfluid cause non-hydrodynamic spin diffusion^18^. This contribution can be made exponentially small in the zero-temperature limit (1000 s life time has been achieved^11^) or dominant at higher temperatures. Observing and controlling the quasi-perpetual time crystal motion inevitably causes also external dissipation,^54^ in our case radiation losses in the measurement circuitry^18^. Both of these dissipation mechanisms cause exponential population decay in time, in combination described by time constant *τ*_*N*_. The time crystals are well defined provided the life time, which here is *τ*_*N*_ ~ 10 s, is much longer than the time it takes for the time crystal to form after the pulse (here *τ*_*E*_ ~ 0.1 s)^5,6^.

The two level system, in the absence of coupling between the states, is described by the “superposition” wave function ${{\Psi }}=b\,{e}^{-i{\omega }_{{{{{{{{\rm{B}}}}}}}}}t}+s\,{e}^{-i{\omega }_{{{{{{{{\rm{S}}}}}}}}}t}$, where $b=\sqrt{{N}_{{{{{{{{\rm{B}}}}}}}}}}{e}^{-i{\varphi }_{{{{{{{{\rm{B}}}}}}}}}}$ and $s=\sqrt{{N}_{{{{{{{{\rm{S}}}}}}}}}}{e}^{-i{\varphi }_{{{{{{{{\rm{S}}}}}}}}}}$. Only the relative phase enters the dynamics of the system. Hence, *b* can be chosen to be real, and the combination *b*, *s* conveniently illustrated by a macroscopic Bloch sphere (Fig. 2c): The surface corresponds to states with total magnon number *N*_0_ = ∣*b*∣^2^ + ∣*s*∣^2^ = *N*_B_ + *N*_S_, and the interior to smaller magnon numbers reached during the population decay. The weights of the basis states in the superposition, that is, the fraction of the total population in the bulk (surface) state is given by the polar angle *θ* with ${N}_{{{{{{{{\rm{B}}}}}}}}}={N}_{0}\cos (\theta /2)$ (${N}_{{{{{{{{\rm{S}}}}}}}}}={N}_{0}\sin (\theta /2)$). The relative phase *ϕ* corresponds to the azimuthal angle in the *x*-*y* plane of the sphere. It evolves in time according to

3 $$\phi ={\varphi }_{{{{{{{{\rm{B}}}}}}}}}-{\varphi }_{{{{{{{{\rm{S}}}}}}}}}+\int\nolimits_{0}^{t}({\omega }_{{{{{{{{\rm{B}}}}}}}}}-{\omega }_{{{{{{{{\rm{S}}}}}}}}}){{{{{{{\rm{d}}}}}}}}t.$$

We note that controlling the relative phase is beyond the scope of the present work and requires adjusting the NMR coil geometry.

### Level dynamics in a flexible trap

^3^He-B is a p-wave superfluid, hence, the orbital momentum of the Cooper pairs is equal to one. In the sample container cylinder, the average orbital momentum **L** is distributed symmetrically (“texture”, Fig. 1) owing to the orienting effects of the magnetic field and the container walls. In addition, we create an axial minimum of **H** using a pinch coil, which confines the magnons due to the Zeeman energy. The bulk trapping potential *U*(**r**) = *U*_**H**_ + *U*_**L**_ therefore has a magnetic part,

4 $${U}_{{{{{{{{\bf{H}}}}}}}}}=\hslash {\omega }_{0}({{{{{{{\bf{r}}}}}}}})\,,$$

and a component created by the **L** distribution owing to the spin-orbit interaction

5 $${U}_{{{{{{{{\bf{L}}}}}}}}}=\hslash \frac{4{{{\Omega }}}_{B}^{2}}{5{\omega }_{0}}{\sin }^{2}({\beta }_{{{{{{{{\bf{L}}}}}}}}}({{{{{{{\bf{r}}}}}}}})/2)\,.$$

Here *ω*_0_(**r**) = ∣*γ***H**(**r**)∣ is the local Larmor frequency which depends on position **r**, Ω_*B*_ is the B-phase Leggett frequency, *γ* ≈ −2⋅10^8^rad s^−1^ T^−1^ is the gyromagnetic ratio of ^3^He, and the order parameter distribution is parametrised by the tipping angle of the orbital anisotropy axis, *β*_*L*_(**r**), measured from the direction of the magnetic field **H**, oriented along the cylinder axis.

Bringing the free surface above the trap centre distorts the order-parameter trap as *β*_**L**_ = 0 at the free surface, creating a local minimum at the surface. Note that we study the time crystals in a frame rotating at the Larmor frequency *ω*_0_ where the uniform magnetic field is absent. Where the notation *ω*_0_ is used without an explicit reference to position, this means Larmor frequency in the middle of the bulk trap, corresponding to the minimum of the harmonic trapping potential. The time crystals located in the two traps can be identified and their frequencies adjusted by changing the profile of the field minimum, aided by the different relaxation rates. Below we concentrate on studying the feedback created by the flexible bulk trap.

The harmonic bulk trap has a radial trapping frequency *ω*_*r*_/(2*π*) ~ 200 Hz corresponding to *U*_**L**_ and an axial trapping frequency *ω*_*z*_/(2*π*) ~ 20 Hz corresponding to *U*_**H**_. The resulting precession frequency is *ω*_0_ + *ω*_*r*_ + *ω*_*z*_/2. Therefore the axial trap can be neglected in the below analysis. It is thus convenient to measure all frequencies in the frame rotating at *ω*_0_. A more detailed analysis of the bulk trap can be found in refs. ^18–20^.

The textural part of the trapping potential feels local magnon density due to spin-orbit interaction: The equilibrium texture minimises a range of free-energy contributions, including the orienting effects of the magnetic field and the sample container walls^55^. An important additional contribution is the spin-orbit interaction energy

6 $${F}_{{{{{{{{\rm{so}}}}}}}}}=| {{\Psi }}({{{{{{{\bf{r}}}}}}}}){| }^{2}{U}_{{{{{{{{\bf{L}}}}}}}}}\,,$$

where ${{\Psi }}({{{{{{{\bf{r}}}}}}}})\propto {\sin }^{2}{\beta }_{{{{{{{{\bf{M}}}}}}}}}/2$ contains the spatial variation of magnon density which gives rise to the feedback effect. That is, the bulk trap profile and the shape of the time crystal wave function depend on *N*_B_ so that d*ω*_B_/d*N*_B_ < 0. In the limit of large magnon number the bulk trapping frequency follows^13^

7 $${\omega }_{{{{{{{{\rm{B}}}}}}}}}({N}_{{{{{{{{\rm{B}}}}}}}}})={\bar{\omega }}_{{{{{{{{\rm{B}}}}}}}}}(1-k{N}_{{{{{{{{\rm{B}}}}}}}}}^{p})$$

Here *k* > 0 depends on the rigidity of the textural trap and the profile of the magnetic field minimum, *p* ≈ 5/7,^13^ and ${\bar{\omega }}_{{{{{{{{\rm{B}}}}}}}}}$ stands for the time crystal trapping frequency in the limit of zero magnons. We emphasise that although *ω*_B_ changes during the decay of the magnon time crystal, the change is very slow as compared with *ω*_0_/(2*π*) ~ 1MHz, and we can thus assume that the wave functions always correspond to the instantaneous trap shape^21^. Note that the surface trap is rigidified by the adjacency of the free surface, and *ω*_S_ is therefore independent of *N*_S_ to a good approximation.

It is possible to describe the self-trapping effect numerically in a self-consistent calculation of the order parameter texture^55,56^, the resulting trap^20^, the time crystal wave function^13,14,21,57^, and population decay^18,19,24,58^. That is however not necessary for understanding the experiments presented in this Article, because finding a general form of Eq. (7) can be circumvented by fitting and numerical differentiation of the experimental data where necessary, and all other effects can be measured independently. For simplicity, we refer to Eq. (7) in the below discussion, but the reader should bear in mind that the general form of the nonlinearity is more complicated.

### Josephson coupling

Let us study the observable consequences of the population oscillation. We use the language of the Josephson effect, analogous to the AC Josephson effect^6^, as the oscillation amplitude can only be reliably extracted from the experiment far from the avoided crossing. Near the avoided crossing one should use the more general Rabi oscillation picture.

The amplitude of the AC Josephson population oscillation is

8 $${{\Delta }}{N}_{{{{{{{{\rm{B}}}}}}}}}=\frac{{{\Omega }}\sqrt{{N}_{{{{{{{{\rm{B}}}}}}}}}{N}_{{{{{{{{\rm{S}}}}}}}}}}}{| {\omega }_{{{{{{{{\rm{B}}}}}}}}}-{\omega }_{{{{{{{{\rm{S}}}}}}}}}| }.$$

Here Ω is the coupling, and Δ*N*_B_ ≡ −Δ*N*_S_. The Josephson frequency is *ω*_J_ = ∣*ω*_B_ − *ω*_S_∣. This oscillation modulates the bulk condensate frequency *ω*_B_ as follows from the self-trapping Eq. (7). The frequency modulation (FM) is sinusoidal to a good approximation. This is because the amplitude of the population oscillation is small as compared with the total population, and Eq. (7) can be linearised.

The resulting instantaneous bulk time crystal frequency ${\tilde{\omega }}_{{{{{{{{\rm{B}}}}}}}}}$ can be written as

9 $${\tilde{\omega }}_{{{{{{{{\rm{B}}}}}}}}}(t)={\omega }_{{{{{{{{\rm{B}}}}}}}}}({N}_{{{{{{{{\rm{B}}}}}}}}})+{{\Delta }}{\omega }_{{{{{{{{\rm{B}}}}}}}}}\cos [({\omega }_{{{{{{{{\rm{B}}}}}}}}}({N}_{{{{{{{{\rm{B}}}}}}}}})-{\omega }_{{{{{{{{\rm{S}}}}}}}}})t].$$

Here Δ*ω*_B_ is the FM amplitude. It is connected to the population oscillation amplitude Δ*N*_B_ by

10 $${{\Delta }}{\omega }_{{{{{{{{\rm{B}}}}}}}}}={{\Delta }}{N}_{{{{{{{{\rm{B}}}}}}}}}\,{{{{{{{\rm{d}}}}}}}}{\omega }_{{{{{{{{\rm{B}}}}}}}}}({N}_{{{{{{{{\rm{B}}}}}}}}})/{{{{{{{\rm{d}}}}}}}}{N}_{{{{{{{{\rm{B}}}}}}}}}.$$

Fourier decomposition of the resulting frequency-modulated signal yields

11 $${A}_{{{{{{{{\rm{B}}}}}}}}}^{(n)}={J}_{n}({{\Delta }}{\omega }_{{{{{{{{\rm{B}}}}}}}}}/({\omega }_{{{{{{{{\rm{B}}}}}}}}}-{\omega }_{{{{{{{{\rm{S}}}}}}}}}))| {e}^{-i({\omega }_{{{{{{{{\rm{B}}}}}}}}}+n({\omega }_{{{{{{{{\rm{B}}}}}}}}}-{\omega }_{{{{{{{{\rm{S}}}}}}}}}))t}|$$

Here *J*_*n*_ is the Bessel function of the first kind of order *n*. The bulk main trace corresponds to *n* = 0. Combining the above expressions, and denoting the first side band (∣*n*∣ = 1) amplitude as *A*_SB_, the coupling term can be linearised and expressed in quantities that can be directly measured:

12 $${{\Omega }}=\frac{2{A}_{{{{{{{{\rm{SB}}}}}}}}}{({\omega }_{{{{{{{{\rm{B}}}}}}}}}-{\omega }_{{{{{{{{\rm{S}}}}}}}}})}^{2}}{{A}_{{{{{{{{\rm{B}}}}}}}}}^{2}{A}_{{{{{{{{\rm{S}}}}}}}}}\,{{{{{{{\rm{d}}}}}}}}{\omega }_{{{{{{{{\rm{B}}}}}}}}}({A}_{{{{{{{{\rm{B}}}}}}}}})/{{{{{{{\rm{d}}}}}}}}{A}_{{{{{{{{\rm{B}}}}}}}}}^{2}}$$

Here we assumed that the filling factors of the bulk and surface states in Eq. (2) are equal and constant. Where the time crystal shapes are changing due to changes in the trap profile, the coupling extracted using the above expression is therefore only approximate.

The side band of the bulk time crystal is seen in Fourier analysis of the experimental signal (Fig. 5d). The coupling extracted from this record using Eq. (12) extrapolates to Ω/(2*π*) ≈ 1.7 Hz at the crossing, in good agreement with the fitted simulation value Ω/(2*π*) ≈ 1.4 Hz. Note that there should be another side band symmetrically at lower frequency than the bulk trace, but it is covered by the surface trace at exactly the same frequency.

The surface trap is only weakly modified in similar fashion, yielding no visible side bands in the experiment. That is, the AC Josephson effect in a fully rigid trap results in no side bands owing to complex interference of the two wave functions. This can be confirmed by solving the dynamics of the rigid non-decaying coupled system analytically. We used this result to test the validity of the numerical simulation discussed below. Note that Ref. ^6^ misleadingly implies that population oscillations directly cause the side bands even in the absence of nonlinear feedback.

Near the avoided crossing one should use the more general Rabi oscillation picture. Solving for the eigenfrequencies of the Hamiltonian in the Rabi regime yields the Rabi frequency ${\omega }_{{{{{{{{\rm{R}}}}}}}}}=\sqrt{{({\omega }_{{{{{{{{\rm{B}}}}}}}}}-{\omega }_{{{{{{{{\rm{S}}}}}}}}})}^{2}+{(2{{\Omega }})}^{2}}$. In the limit Ω ≪ ∣*ω*_B_ − *ω*_S_∣ this is reduced to *ω*_R_ = *ω*_J_. The region where *ω*_R_ ≠ *ω*_J_ is not directly visible in the experiments due to interference effects.

### Landau–Zener tunnelling

In the presence of exponential population dissipation, the time crystal population follows

13 $${N}_{\alpha }(t)={N}_{\alpha }(t=0){e}^{-2t/{\tau }_{\alpha }},$$

where 1/*τ*_*α*_ is the relaxation rate of the measured signal (2), and *α* is either B for the bulk or S for surface. For the surface time crystal this makes little difference other than that the population decays slowly. The bulk time crystal frequency *ω*_B_ depends on *N*_B_ according to Eq. (7), and the frequency therefore increases during the decay. Hence, we have obtained the flexible two-level system described by the Hamiltonian (1).

Let us choose *ω*_B_(*N*_B_ = 0) > *ω*_S_ and *N*_B_(*t* = 0) such that *ω*_B_(*N*_B_(*t* = 0)) < *ω*_S_. Now the frequencies of the surface and bulk time crystals will cross in the eigenbasis where Ω = 0. If Ω > 0 and *N*_B_ decreases adiabatically, magnons in the bulk trap will smoothly move to the surface trap, remaining in the global ground state in an avoided crossing. The minimum frequency separation of the global ground state and the excited state at the avoided crossing is 2 Ω, as can be solved from the Hamiltonian.

If the avoided crossing is passed non-adiabatically, a part of the ground state magnon population moves to the excited state. This phenomenon is known as the Landau–Zener–Stueckelberg–Majorana effect. In our case this means that after the avoided crossing some population remains in the bulk trap, which corresponds to the new excited state in the system. The fraction of population promoted to the excited state is^59^

14 $$\delta n=\exp \left(-\frac{2\pi {{{\Omega }}}^{2}}{| {\partial }_{t}({\omega }_{{{{{{{{\rm{B}}}}}}}}}-{\omega }_{{{{{{{{\rm{S}}}}}}}}})| }\right),$$

where ∂_*t*_ stands for time derivative. Note that while in the canonical Landau-Zener problem the time derivative is constant, in our case it keeps changing. However, the magnitude of the Landau-Zener population transfer is determined within a time window $\sim\! 1/\sqrt{| {\partial }_{t}({\omega }_{{{{{{{{\rm{B}}}}}}}}}-{\omega }_{{{{{{{{\rm{S}}}}}}}}})| }$ of the level crossing^23^ (≲100 ms wide in our experiment). Therefore, *ω*_B_(*t*) can be linearised, or in other words, the time derivative taken at the avoided crossing gives the correct Landau-Zener population transfer.

### Numerical simulation

The time crystal two-level Hamiltonian can be combined with the slow decay into a pair of equations:

15 $$i{\partial }_{t}{{{\Psi }}}_{{{{{{{{\rm{B}}}}}}}}}=({\omega }_{{{{{{{{\rm{B}}}}}}}}}({N}_{{{{{{{{\rm{B}}}}}}}}})-i\,{\tau }_{{{{{{{{\rm{B}}}}}}}}}^{-1}){{{\Psi }}}_{{{{{{{{\rm{B}}}}}}}}}-{{\Omega }}{{{\Psi }}}_{{{{{{{{\rm{S}}}}}}}}}$$

16 $$i{\partial }_{t}{{{\Psi }}}_{{{{{{{{\rm{S}}}}}}}}}=({\omega }_{{{{{{{{\rm{S}}}}}}}}}-i\,{\tau }_{{{{{{{{\rm{S}}}}}}}}}^{-1}){{{\Psi }}}_{{{{{{{{\rm{S}}}}}}}}}-{{\Omega }}{{{\Psi }}}_{{{{{{{{\rm{B}}}}}}}}}.$$

Here the right hand side corresponds to the Hamiltonian (1), and *i* is the complex unit. This pair of equations can be solved numerically. Our main motivation for the numerical simulation is to show that the simple two-level Hamiltonian describes the dynamics of the system exhaustively, that is, that the large population transfer is explained by the intrinsic dynamics of the two-level Hamiltonian. The most important test for this picture is the avoided crossing and the related population transfer. Reproducing the Rabi oscillations that result in a frequency-modulation of the time crystal frequencies is a secondary test.

The initial time crystal wave functions, the time crystal decay rates, and the bulk trap self-trapping power law, Eq. (7), can be extracted from experimental data independently and used as the parameters of the numerical simulation. The coupling Ω at the avoided crossing cannot be directly extracted from the experiment, and is used as a fitting parameter. To compare with the measured signal we also need the filling factors *c*_*α*_. They are used as fitting parameters as well.

We note that to reproduce the experimental signals in general, three additional effects need to be included in the simulation: (i) The surface time crystal frequency depends on the population in the bulk trap and (ii) also on the population in the surface trap; *ω*_S_ = *ω*_S_(*N*_B_, *N*_S_). (iii) Both *τ*_B_ and *τ*_S_ change at the avoided crossing, and to allow for this transition to take place smoothly, we use a smooth interpolation function between the asymptotic relaxation rates in the Rabi regime with the width of the crossing region being another fitting parameter with value ~Ω^−1^. Effect (i) is due to the widening of the constriction that separates the time crystals (Fig. 2). This connection is included in the simulation, and it is seen as a decrease of *ω*_S_ in Fig. 4a at *t* < 1 s, where bulk population is large and surface population negligible. However, this effect can be safely neglected in the analysis of the Landau–Zener effect in Fig. 3, because the avoided crossing takes place at small *N*_B_. The second dependence (ii) is what produces the frequency-oscillations of the magenta line in Fig. 4c. Both effects can be extracted from experimental data independently.

## Data Availability

The data used in this study are available in the Zenodo database under accession code 10.5281/zenodo.6510863.

## Code availability

The simulation codes and guidance in their use are available from the corresponding author upon reasonable request.
