# Publisher Correction: Nonlinear two-level dynamics of quantum time crystals

**DOI:** 10.1038/s41467-022-31647-z

**Published:** 2022-07-02

**Authors:** S. Autti, P. J. Heikkinen, J. Nissinen, J. T. Mäkinen, G. E. Volovik, V. V. Zavyalov, V. B. Eltsov

**Affiliations:** 1 Low Temperature Laboratory, Department of Applied Physics, Aalto University, POB 15100, FI-00076 Aalto, Finland; 2 Department of Physics, Lancaster University, Lancaster, LA1 4YB UK; 3 Department of Physics, Royal Holloway University of London, Egham, Surrey TW20 0EX UK; 4 L.D. Landau Institute for Theoretical Physics, Moscow, Russia

**Keywords:** Quantum fluids and solids, Magnetic properties and materials, Quantum mechanics, Bose-Einstein condensates

Correction to: *Nature Communications* 10.1038/s41467-022-30783-w, published online 2 June 2022.

The original version of this Article contained an error in Equation 1 in the PDF version. The right hand side was written as a single matrix element instead of a two-by-two matrix, and incorrectly read:

$${\mathcal H} =\hslash ({\omega }_{{{{\rm{B}}}}}[{N}_{{{{\rm{B}}}}}(t)]-\Omega -\Omega {\omega }_{{{{\rm{S}}}}}).$$

The correct form of Equation 1 is:

$${\mathcal H} =\hslash \left(\begin{array}{ll}{\omega }_{{{{\rm{B}}}}}[{N}_{{{{\rm{B}}}}}(t)] & -\Omega \\ -\Omega & {\omega }_{{{{\rm{S}}}}}\end{array}\right).$$

This has been corrected in the PDF version of the Article.

